# Effect of Kisspeptin-Type Neuropeptide on Locomotor Behavior and Muscle Physiology in the Sea Cucumber *Apostichopus japonicus*

**DOI:** 10.3390/ani13040705

**Published:** 2023-02-17

**Authors:** Xueying Guo, Libin Zhang, Kang Xiao

**Affiliations:** 1 CAS Key Laboratory of Marine Ecology and Environmental Sciences, Institute of Oceanology, Chinese Academy of Sciences, Qingdao 266071, China; 2 Laboratory for Marine Ecology and Environmental Science, Qingdao National Laboratory for Marine Science and Technology, Qingdao 266237, China; 3 Center for Ocean Mega-Science, Chinese Academy of Sciences, Qingdao 266071, China; 4 CAS Engineering Laboratory for Marine Ranching, Institute of Oceanology, Chinese Academy of Sciences, Qingdao 266071, China; 5 Shandong Province Key Laboratory of Experimental Marine Biology, Qingdao 266071, China; 6 University of Chinese Academy of Sciences, Beijing 100049, China; 7 Beijing Yanshan Earth Critical Zone National Research Station, College of Resources and Environment, University of Chinese Academy of Sciences, Beijing 101408, China

**Keywords:** *Apostichopus japonicus*, kisspeptin-type neuropeptide, locomotion, longitudinal muscle, metabolic physiology

## Abstract

**Simple Summary:**

The sea cucumber *Apostichopus japonicus* is an important aquatic invertebrate, which has high nutritional and medicinal value. Kisspeptins are neuropeptides encoded by the *kiss1* gene, and little is known about them outside of the vertebrate lineage. In this study, we investigated the effect of KPs on locomotor behavior in one control group and two treatment groups (AjK1 and AjK2). We found that AjK1 had a significant dose effect, mainly by reducing the stride length and duration of movement to decrease the movement distance of sea cucumbers, whereas AjK2 had little inhibitory effect at the same dose. The levels of phosphatidylethanolamine (PE), phosphatidylcholine (PC),uridine, glycine, and L-serine in the longitudinal muscle of *A. japonicus* treated with AjK1 differed significantly from those of the control, which may explain the observed changes in locomotor behavior.

**Abstract:**

Kisspeptins are neuropeptides encoded by the *kiss1* gene, and little is known about them outside the vertebrate lineage. Two kisspeptin-type neuropeptides (KPs) have been discovered in *Apostichopus japonicus* (AjK1 and AjK2), an edible sea cucumber, and have been linked to reproductive and metabolic regulation. In this study, we evaluated how KPs affected locomotor behavior in one control group and two treatment groups (AjK1 and AjK2). We discovered that AjK1 had a significant dose effect, primarily by shortening the stride length and duration of movement to reduce the sea cucumber movement distance, whereas AjK2 had little inhibitory effect at the same dose. The levels of phosphatidylethanolamine (PE), phosphatidylcholine (PC), uridine, glycine, and L-serine in the longitudinal muscle of *A. japonicus* treated with AjK1 differed significantly from those of the control, which may explain the observed changes in locomotor behavior. Treatment with AjK2 induced changes in aspartate levels. Our results imply that AjK1 is more likely than AjK2 to have a role in the regulation of *A. japonicus* locomotion.

## 1. Introduction

Neuropeptides are endogenous peptides synthesized in nerve cells. They, by and large, comprise 1–300 amino acids, and they assume significant parts in cell correspondence, homeostasis, behavior, and physiological processes [1,2]. Current evidence proves that all neuropeptides are derived from larger precursor proteins that are processed and modified by enzymes during the time spent in transport to ultimately generate neuropeptides with bioactivities [2,3]. Neuropeptides can go about as neurotransmitters or modulators locally in the nervous system or as neurohormones that are moved through the circulatory system to act on distant target cells [2,4,5]. Neuropeptides are ancient signaling molecules whose origins can be traced back to a common ancestor in bilaterians [6].

The hypothalamic neuropeptide kisspeptins, otherwise called metasti, have a C-terminal end that contains the common Arg-Phe-amide motif, and they have a place with the RFamide-type neuropeptide families [6,7,8]. Kisspeptin-type neuropeptides (KPs) encoded by the *kiss1* gene (melanoma metastasis-suppressor gene) are endogenous ligands of G-protein-coupled receptor 54 (GPR54, likewise called AXOR12 or hOT7T175) [8,9]. Kisspeptins and GPR54 together make up the kisspeptin/GPR54 system, which can affect the development and metastasis of cancer cells, partake in reproductive regulation, and influence the endocrine system [10]. Based on these functions, kisspeptins and their structural analogs and antagonists have become a research hotspot in the field of animal neuroendocrinology. The kisspeptin neuropeptide signaling system regulates a variety of metabolic parameters, including body weight and energy expenditure, food intake, respiratory rate, locomotor activity, and glucose metabolism, yet few studies related to kisspeptin neuropeptide in invertebrates have been reported [7,11].

Echinoderms are phylogenetically intermediate between chordates and protostomes, and they have a special evolutionary status as well as unique biological characteristics. These traits make them an important model for the study of neuropeptide signaling systems and functions, and a focus of biological evolutionary research, which can provide key support for the evolution of neuropeptide signaling systems and comparative physiological studies [3,12]. The sea cucumber *Apostichopus japonicus*, which lives in the northwest Pacific Ocean, is a mariculture species with high nourishing and monetary worth [13]. To date, two putative KPs have been reported in *A. japonicus* in light of kisspeptin-type precursors: a 32-amino-acid peptide with a disulfide bond (AjK1) and an 18-amino-acid peptide (AjK2) [7,14]. AjK1 and AjK2 may be involved in the regulation of ensuing physiological processes by activating their receptors, AjKissR1/AjKissR2 [7]. Wang et al. (2020) suggested that the kisspeptin system participates in the regulation of metabolic equilibrium and seasonal reproduction of *A. japonicus* [7]. The broad distribution and different expression levels of AjKissR1 and AjKissR2 in multiple tissues, including longitudinal muscle, suggest that KPs may act on muscle tissue and that these two receptors and their corresponding neuropeptides may have different functions [7]. An organism’s locomotion behavior is involved in predation, escape, and migration [15,16]. Sea cucumber locomotion is primarily realized by alternate contraction and relaxation of longitudinal muscle [17,18]. Existing reports demonstrate that neuropeptides assume a crucial part in locomotor activity [19]. Based on the expression distribution of KP receptors in the longitudinal muscle of *A. japonicus*, we speculated that neuropeptides that specifically activated AjKissR1 and AjKissR2 may not just affect the locomotion of *A. japonicus*, and they also may have different effects.

Metabonomics is a typical omics technique used to identify the concentration of little molecular substances in tissues [20]. Liquid chromatography-tandem mass spectrometry (LC-MS/MS) is one of the most utilized technologies because of its intrinsic specificity, sensitivity, and speed [21]. This technique has been effectively applied to the physiological appraisal of muscle metabolism of sea cucumbers [1,22]. According to the point of view of neuroendocrinology and through intraperitoneal injection of kisspeptins, we explored the impacts of AjK1 and AjK2 on locomotor behavior and the regulation of muscle metabolites and metabolic pathways of *A. japonicus* using time-lapse photography, behavioral analysis software, combined pharmacokinetics, and LC-MS/MS technology. Our results helped fill the hole in information about KPs in invertebrates and could be applied to understanding the evolutionary origin of animal neuropeptides and peptide hormones.

## 2. Materials and Methods

### 2.1. Laboratory Animals and Rearing Environment

Sea cucumbers *A. japonicus* used in this study were obtained from the port of Zhuwang, Laizhou, China (37° N, 119° E). They were put in rectangular aquariums (3 m × 2 m) filled with filtered and oxygenated seawater (TEMP 14.8 ± 0.6 ºC, SAL 29 psu, DOC > 7.5 mg/L). These sea cucumbers were fed a homemade diet consisting of sea mud plus 30% *Sargassum* powder. The feces and remaining feed were eliminated with a hose every day. The sea cucumbers were cultured under these conditions until used in the experiment.

### 2.2. Neuropeptide Synthesis

The sequences of AjK1 and AjK2 used in this study were reported by Wang et al. (2020) and were predicted in silico from *A. japonicus* transcriptome data, which were AGSLDCLEASCEDVERRGRQPNRNAHYRTLPF-NH_2_ and SAVKNKNKSRARPPLLPF-NH_2_, individually [7]. Both AjK1 and AjK2 were verified as C-terminally amidated peptides by mass spectrometry analysis, and two cysteine residues of AjK1 were linked by a disulfide bridge [7,14]. The peptides were synthesized using a solid-phase synthesis method by APeptide Co., Ltd. (Shanghai, China). The molecular mass of AjK1 was 3659.01 g/mol, and its purity was 96.80%. The molecular mass of AjK2 was 2022.40 g/mol, and its purity was 96.61%. AjK1 and AjK2 were put away at −20 ºC before use.

### 2.3. Locomotion Behavior Video Capture

For the behavioral experiments, we divided 56 sea cucumbers in good condition into seven groups (*n* = 8 sea cucumbers/group, 99.41 ± 13.06 g). They were separated into one control group, three AjK1 treatment groups (K1), and three AjK2 treatment groups (K2). In the seawater control group (S), 0.1% (v/w) natural seawater that had been sterilized at a high temperature and in an autoclave was injected into the coelom of the animals. Sea cucumbers in the treatment groups were injected with the same volume as the S group, but with the KP solution rather than saltwater. The test concentrations of AjK1 and AjK2 were determined at 10 μM (high, H), 1 μM (medium, M), and 0.1 μM (low, L) based on Ding et al. (2019), Kato et al. (2009), and other laboratory research (unpublished data) [1,23]. AjK1 and AjK2 were dissolved in sterilized seawater and diluted to these levels. Information about the experimental groups is provided in Table 1.

Each experimental sea cucumber was photographed separately in a white plastic basin (70 cm in diameter, water line 7 cm) to make it more straightforward for the analysis software to recognize individuals. A TLC200 time lapse video camera (Brinno, Taiwan, China) was mounted on a camera stand over each basin, and a 5 W bulb positioned above the basin provided brightening (Supplementary Figure S1). Before the start of the behavioral experiment, the experimental sea cucumbers were kept in the shooting environment for at least 24 h to acclimatize to the circumstance [1]. This experiment was conducted under continuous light source (12L:0D). Both before and after neuropeptide administration, the behavioral recording time was 12 h.

### 2.4. Data Analysis of Locomotion Behavior

EthoVision XT software (version: 10.1, Noldus Inc., Wageningen, Netherlands) was used to analyze the behavioral videos by calculating distance, moving duration, and mean velocity. Successive alternations of contraction and relaxation caused sea cucumbers to move ahead [18]. Therefore, we characterized one step as one contraction and relaxation, and stride length was the distance moved per step. By counting steps, we calculated the average stride length and stride frequency. The difference value of a behavioral parameter (ΔY) was obtained by subtracting the post-injection value from the pre-injection value, and it represented the influence of the KP on locomotor behavior of *A. japonicus* (ΔY > 0 inhibition, ΔY < 0 promotion).

We assumed that the effect of the KPs on *A. japonicus* satisfied the first-order kinetic model [24]:

ΔY = A _max_ · e^−kt^

We obtained the maximum effect parameter A_max_ and decay rate constant k with this equation. We obtained the half-life τ as follows:

τ = ln2/k

We analyzed the locomotion data using one-way analysis of variance (ANOVA), pared-samples *t*-test, two-sided *t*-tests, and nonlinear regression analysis in SPSS 27 software (IBM, Armonk, NY, USA).

### 2.5. Muscle Metabolomics

For this experiment, we divided 48 healthy sea cucumbers into a control group (CM), an AjK1 treatment group (K1M), and an AjK2 treatment group (K2M), each containing 16 animals (*n* = 16 sea cucumbers/group, 92.11 ± 11.25 g). For the two treatment groups, the neuropeptide concentration was 10^–5^ M. The injection amount of KPs was 0.1% of the body weight (v/w) of *A. japonicus*. After neuropeptide treatment, about 2 g of muscle tissue were taken from the body wall of each sea cucumber. In vitro tissues were cleaned with ultrapure water, dried with absorbent paper, placed in sterile cryopreservation tubes, and stored at –80 °C. Two samples were combined into one tube for metabolomics detection.

Muscle metabolomics, including metabolite extraction, LC-MS/MS analysis, and bioinformatics analysis, was carried out in collaboration with Guangzhou Genedenovo Biotechnology Co., Ltd. (Guangzhou, China). The detailed experimental procedures and methods are consistent with those of Li et al. (2019) [25].

## 3. Results

### 3.1. Changes in Locomotion of A. japonicus after KPs Injection

**Figure 1:**
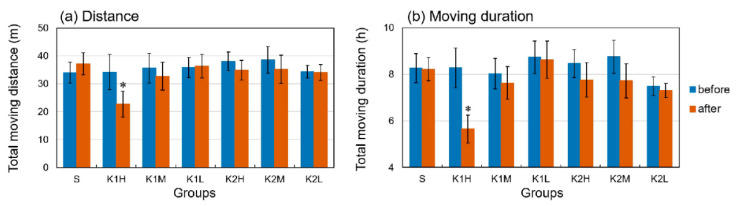
The total distance moved (**a**) and cumulative moving duration (**b**) before and after injection for each group. The data in the figure are total measured data for 12 h. Each error bar represents the standard error. S is the seawater control group, K1 and K2 are the neuropeptide treatment groups, and H/M/L represent high, medium, and low concentrations. *, significant difference within the group (*p* < 0.05).

**Figure 2:**
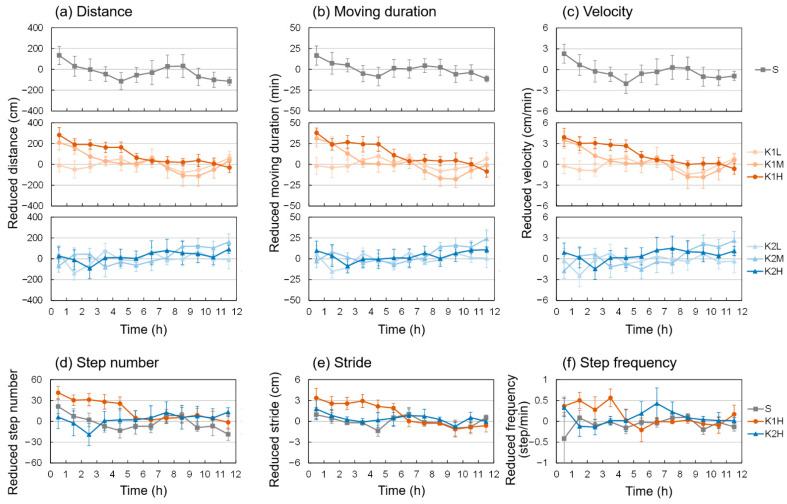
Trend of locomotor behavior of *A. japonicus* within 12 h after injection compared with before injection: (**a**) distance; (**b**) moving duration; (**c**) velocity; (**d**) step number; (**e**) stride length; (**f**) step frequency. Behavioral data were measured hourly, and the values in the figure are the differences (ΔY) at the same moment in the two adjacent days before and after injection. The error bars in the figure represent the standard error (*n* = 8 for each group). S is the seawater control group, K1 and K2 are the neuropeptide treatment groups, and H/M/L represent high, medium, and low concentrations.

Figure 1 shows the total distance and moving duration of *A. japonicus* under different treatments. Comparative with the control group, the total distance (moving duration) in the K1H group decreased after injection from 34.57 ± 17.39 m to 23.34 ± 12.84 m (from 8.36 ± 2.25 h to 5.75 ± 1.64 h), a reduction of about 30%, and the within group and between group comparisons showed significant difference; however, no apparent changes or significant differences were detected in any of the other groups. Figure 2 shows changes in inhibition of locomotion over time (0–12 h) for the AjK1 and AjK2 treatment groups at different doses (L/M/H). High doses of AjK1 had inhibition effects, especially in the first five hours after injection. The K2H group showed a reduction that lasted 2–3 h, but any influence of the other treatment groups was not obvious.

**Figure 3:**
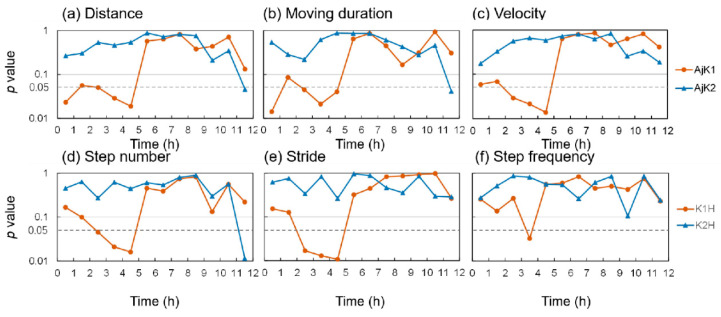
Significance of differences between different neuropeptide groups changed over time: (**a**) distance; (**b**) moving duration; (**c**) velocity; (**d**) step number; (**e**) stride length; (**f**) step frequency. (**a**–**c**) show the difference between different dose levels (0/L/M/H) of K1 (or K2), which were compared by one-way ANOVA. (**d**–**f**) The difference between the two high concentration treatment groups (K1H and K2H) and the seawater control group, which were compared using *t*-tests (two-tailed). The significance level was set at 0.05.

Next, we compared differences in the locomotor behavior of sea cucumbers treated with different KPs and doses. To analyze the distance, moving duration, and mean velocity, we used one-way ANOVA for each KP, with concentration set as the factor level. For step number, average stride length, and step frequency, we used two-tailed *t*-tests to compare the distinction between K1H or K2H and S. For the first three parameters, AjK1 had a significant dose impact in the first 5 h; for the latter three parameters, the significance of K1H in the first 5 h was mainly reflected in step number and stride length and was weak in step frequency (Figure 3). In contrast, AjK2 treatment had almost no significant effect throughout the entire experiment.

Then, we analyzed contrasts in locomotor behavior of sea cucumbers treated with various KPs and dosages. To examine the distance moved, cumulative duration of movement, and mean velocity data, we involved one-way ANOVA for every KP, with concentration set as the factor level. For step number, average stride length, and step frequency data, we utilized two followed t-tests to look at the distinction between K1H or K2H and S. For the initial three parameters, AjK1 had a significant dose effect in the initial 5 h; for the last three parameters, the significance of K1H in the first 5 h was predominantly reflected in step number and stride length and was weak in step frequency (Figure 3). Conversely, AjK2 treatment had no massive impact throughout the entire investigation.

AjK1 had a dose effect, and its effectiveness was time dependent. According to the principle of pharmacokinetics, among the steps of absorption, distribution, metabolism, and elimination, we focused on the elimination process because we had directly injected the treatment (i.e., absorption was instantaneous) [24]. We used the first-order kinetic model to fit the A_max_ and k, and τ was obtained by conversion. Table 2 clearly shows the dose response and pharmacokinetics of AjK1. Both high and medium concentrations of AjK1 could fit the kinetic parameters, but the low dose of AjK1 could not be fitted due to the insignificant effect. A_max_ of K1H and K1M were similar, but k and τ were different. The k values of K1H and K1M were about 0.25–0.3 h^–1^ and 0.5–0.6 h^–1^, respectively, and their half-lives were about 2.5–3 h and 1.1–1.2 h, respectively. For K2H, the only parameter that could be fit by the model was stride length, and both A_max_ and τ were lower than those of AjK1. This indicated that at the same dose, the effect of AjK2 was far less than that of AjK1 and that AjK2 had no significant effect on the locomotor behavior of *A. japonicus*.

### 3.2. KPs Induced Changes in Muscle Metabolism

**Figure 4:**
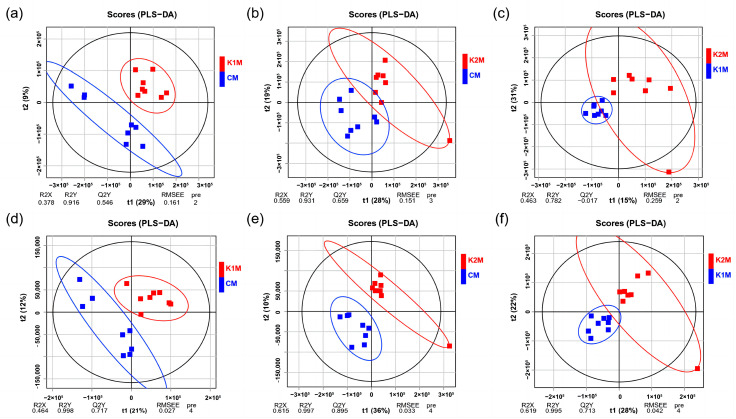
PLS-DA scores plot of longitudinal muscle metabolite profiling. (**a**) CM (in blue) vs. K1M (in red) in positive ion mode, *Q^2^* = 0.546; (**b**) CM (in blue) vs. K2M (in red) in positive ion mode, *Q^2^* = 0.659; (**c**) K1M (in blue) vs. K2M (in red) in positive ion mode, *Q^2^* < 0 (unreliable); (**d**) CM (in blue) vs. K2M (in red) in negative ion mode, *Q^2^* = 0.717; (**e**) CM (in blue) vs. K2M (in red) in negative ion mode, *Q^2^* = 0.895; (**f**) K1M (in blue) vs. K2M (in red) in negative ion mode, *Q^2^* = 0.713.

In this study, we used LC-MS/MS technology to identify metabolites in the muscle tissues of sea cucumbers from the three groups (CM, K1M, K2M). Metabolomics ordinarily uses QC samples for quality control analysis to obtain reliable data [26]. In our study, QC samples were well aggregated in the principal component analysis score plot, which demonstrated that the metabolomic assay results we obtained were dependable (Supplementary Figure S2). The metabolome data for the muscle samples from the three experimental groups were compared by PLS-DA in pairs to obtain the differential metabolic profiles (Figure 4). Prescient capacity (*Q^2^*) values are the most recognized parameter to characterize the reliability of PLS-DA models and *Q^2^* > 0.5 indicated that this model predicted reliably in our study. PLS-DA score plots in both positive and negative ion modes showed relatively obvious differentiation between each of the pairs of groups, which indicated that both AjK1 and AjK2 could induce metabolism changes and that the changes evoked by the two KPs may differ.

### 3.3. Differential Metabolites and Metabolic Pathways under KPs Treatment

**Figure 5:**
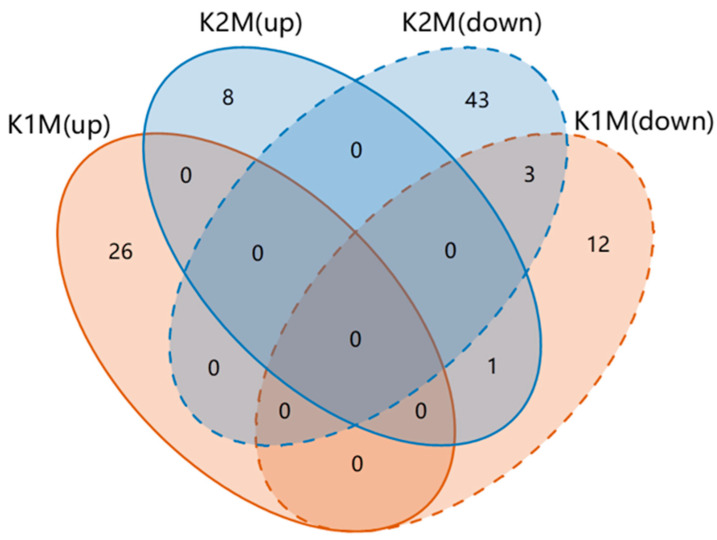
Venn diagram of all differential metabolites from the experimental groups (K1M and K2M) both in positive and negative ion mode. Circles in orange indicate the AjK1 treatment group; circles in blue indicate the AjK2 treatment group; solid lines mean that metabolites are up-regulated; dashed lines mean that they are down-regulated.

**Figure 6:**
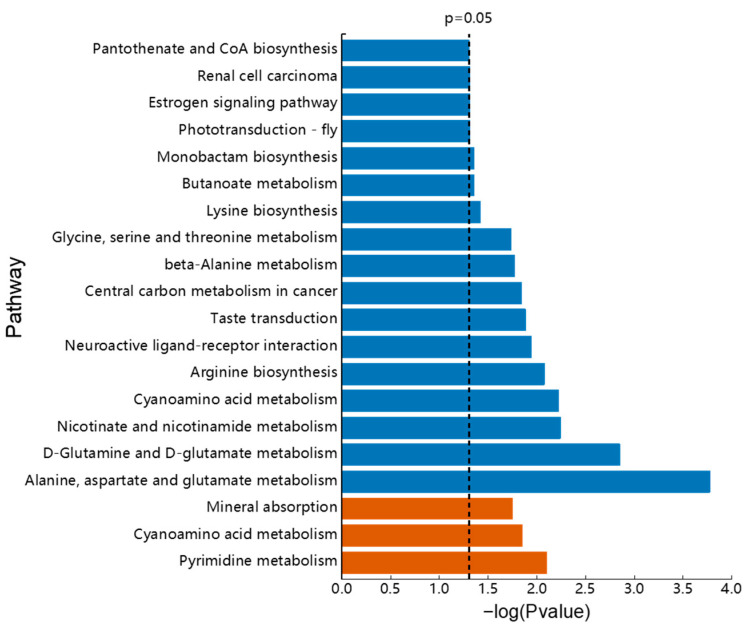
Enrichment analysis of differential metabolic pathways. K1M is shown in orange and K2M is shown in blue. The vertical line mean *p* = 0.05.

We focused on the metabolic differences between the treatment groups and the seawater group and looked at the metabolic differences between the two KP groups. Metabolites with significant changes were identified based on VIP > 1 and *p* < 0.05 (Supplementary Table S1). K1M and K2M were compared with CM in two ion modes and 42 and 55 differential metabolites were detected, respectively. Treatment with AjK1 resulted in up- and down-regulation of 26 and 16 differential metabolites, respectively, while 9 and 46 up- and down-regulated divergent metabolites were found in the AjK2 treatment group. The shared and specific differential metabolites in the different comparison sets are displayed in Figure 5. Only four metabolites were shared by the two treatment groups; however, the two neuropeptides regulated methyl 2-propenyl selenide in opposite directions. Overall, the two KPs had different effects. Enrichment analysis showed that the differential metabolites were involved in numerous metabolic pathways. Treatment with AjK1 and AjK2 significantly interfered with 3 and 17 of them, respectively, most of which involved the metabolism of amino acids (Figure 6, *p* < 0.05).

## 4. Discussion

### 4.1. KPs More Likely to Inhibit Locomotion of A. japonicus

The experimental results indicated that AjK1 was more likely to be involved in locomotor regulation, and AjK2 seemed not to have an effect. Although the movement pattern of *A. japonicus* consisted mainly of random movement, locomotor behavior was inhibited in a dose-dependent manner for the first 5 h after injection of AjK1, whereas AjK2 at the same concentration had little impact. The fundamental factors that affect movement are moving duration, step frequency, and stride length. In our study, the significance of step frequency was weak, suggesting that AjK1 was more likely to inhibit step length and moving duration and increase immobility time. The A_max_ values of K1H and K1M were similar, but the k and τ values differed. The τ of K1H was about 2.5–3 h, which might explain the significant dose effect that occurred within 5 h. After 5 h, the effect disappeared and locomotor behavior returned to the background level. The similar A_max_ values of K1H and K1M may indicate that 1 μM of AjK1 is enough to reach the limit of the inhibitory effect, and increasing the dose only prolongs the action time. It is worth mentioning that the stride of sea cucumbers in the K2H group showed a slight fluctuation, and enhancing the concentration of AjK2 might show the effect more clearly. In addition, there is a circadian rhythm in the locomotion of *A. japonicus*, and the most important factor influencing it is the change in light rhythm. Under continuous light conditions (24L:0D), there were some ups and downs, but the motor behavior rhythm of *A. japonicus* almost disappeared, which was consistent with the results of S group in this experiment [27]. Therefore, we chose to conduct our experiments at night using only uniform experimental light sources and a fixed experimental time, and we used ΔY values for data analysis to minimize the effect of circadian rhythms. In other words, we were concerned with the effect of the injected neuropeptide on the locomotion of *A. japonicus* rather than whether the neuropeptide acted differently on *A. japonicus* during the day and night. Therefore, the effects of circadian rhythms in *A. japonicus* are not addressed in our discussion.

Most studies of locomotor behavior under the influence of KPs have focused on rats and mice, and little is known about their effects on other vertebrates and invertebrates. Kisspeptin-expressing neurons signaling in the arcuate nucleus of the hypothalamus promote physical activity during the dark or active phase of adult female mice [28]. Mammalian kisspeptin-13 induces an increase in both spontaneous and exploratory locomotion in male Sprague–Dawley rats [29], whereas kisspeptin-8 (n-terminally truncated octapeptide) decreases spontaneous movement in adult male Wistar rats [30]. Kissorphin, a new peptide derived from kisspeptin-10, attenuates acute hyperkinesis and sensitization induced by morphine and ethanol in male mice [31]. Although the effects of different KPs on locomotor behavior might differ among different species, our results demonstrated that AjK1 can inhibit the locomotion of *A. japonicus*.

Current research results indicate that multiple neurohormones and neuropeptides are associated with the regulation of locomotor behavior. Ding et al. (2019) reported that melatonin could reduce its exercise endurance and movement efficiency [1]. In contrast, L-type SALMFamide neuropeptide enhanced the exercise endurance and movement efficiency of *A. japonicus* [32], and pedal peptides could enhance exercise endurance and reduce movement efficiency [17]. Kahsai et al. (2010) reported that neuropeptides expressed in the insect brain central complex are involved in the regulation of locomotor behavior [33]. They found that tachykinin could regulate the spatial orientation of *Drosophila melanogaster*. Cui er al. (2020) found that short neuropeptide F (sNPF) had suppressive effects on *D. melanogaster* locomotor activity levels, and down-regulation of sNPF increased the long-distance walking and movement velocity of female fruit flies [34]. NPF also is involved in the locomotor plasticity of locusts and attenuates phase-related locomotor activity [35]. Diaz et al. (2019) identified allatostatin-C as a novel neuropeptide that could mediate evening locomotor activity under different photoperiods in *Drosophila*, and Kiss et al. (2013) reported that drosulfakinin increased *Drosophila* larval locomotion activity and induced an escape response [36,37]. The lack of neuropeptide pigment-dispersing factor can influence the normal locomotor activity of the roundworm *Caenorhabditis elegans* [38]. Neuropeptides such as RYamide, DLamide, and FMRFamide regulate ciliary beat frequency and affect the swimming depth of *Platynereis* larvae [39]. Other reports have shown that neuropeptides such as corticotropin-releasing hormone, thyrotropin-releasing hormone, or orexin affect not only feeding behavior but also locomotor activity in goldish (*Carassius auratus*) [40,41,42]. Intracerebroventricular injections of orexin also can promote locomotion in mice, rats, and dogs, but it has a mild sedative effect on zebrafish (*Danio rerio*) and can inhibit its locomotion [43,44,45,46]. Neuropeptide S, which activates locomotion and reduces anxiety, reinstates extinguished cocaine-seeking behavior in adult male mice via corticotropin-releasing factor receptor 1 [47].

In conclusion, numerous studies have demonstrated that neuropeptides have an impact on locomotor activity. We found that AjK1 can regulate locomotor behavior by inhibiting stride length and moving duration of *A. japonicus*, and reduce their exercise endurance and movement efficiency. This discovery provides new evidence for the involvement of KPs in regulating locomotor behavior.

### 4.2. Mechanisms by Which AjK1 Inhibits Locomotor Behavior of A. japonicus

We used LC-MS/MS to qualitatively and quantitatively analyze the low-molecular-weight metabolites in three groups of muscle samples of *A. japonicus* (CM, K1M, and K2M). The two KPs had obvious interference effects on muscle metabolites (Figure 4). The K1M and K2M groups shared only four differential metabolites, and they did not share the same differential metabolic pathways. Thus, despite the fact that both AjK1 and AjK2 were KPs, we hypothesized that their impacts on the muscle metabolism of *A. japonicus* were distinct. AjK1 was more likely to be involved in regulating the locomotor behavior in *A. japonicus*, according to behavioral data. We assume that the locomotor behavior of *A. japonicus* is inhibited by the significantly altered metabolites and metabolic pathways found in the K1M group. Pyrimidine metabolism, cyanoamino acid metabolism, and mineral absorption are the metabolic pathways in the longitudinal muscle that were significantly impacted by the administration of AjK1. These pathways involved differential metabolites such as phosphatidylethanolamine (PE), phosphatidylcholine (PC), uridine, glycine, L-serine, etc.

The two most important phospholipids in eukaryotic cells are PE and PC [48]. They are the main components of cell membranes, accounting for 50% and 20–30% of total phospholipids, respectively [49,50]. Deletion of muscle-specific PC- and PE-related enzymes leads to decreased synthesis of PE and an increased PC:PE ratio, which are accompanied by decreased skeletal muscle mass and low locomotion [51,52,53]. Therefore, the PC:PE ratio is probably related to skeletal muscle growth and contraction, motor performance, and behavioral plasticity [49,54]. We found that PC18:2(9Z,12Z)/18:0), PC(24:1(15Z)/14:1(9Z)), and PE(P-18:1(11Z)/22:5(4Z,7Z,10Z,13Z,16Z)) were significantly up-regulated after AjK1 administration and that the PC:PE ratio was elevated. This may not only support previous research but also explain the experimental results. The regulation of phospholipids and its effect on muscle contractility may explain the change in stride length after AjK1 injection in *A. japonicus.* Thus, the change in the PC:PE ratio may be the underlying physiological mechanism of AjK1 inhibition of the locomotor behavior of *A. japonicus*.

The pyrimidine metabolic pathway not only maintains suitable pyrimidine levels but also generates bioactive intermediate metabolites [55]. For example, uridine is involved in synthetizing RNA, biofilm RNA, and biological membranes, and the concentration of uridine in human plasma is much higher than that of other purines and pyrimidines [56]. A mixture containing docosahexaenoic acid and/or eicosapentaenoic acid and uridine or its equivalents can reduce locomotor activity to help people rest well at night [57]. Honda et al. (1984) reported that after intraventricular injection of 1 nmol of uridine, locomotor and sleep-waking rhythms became irregular in rats [58]. Uridine also was found to attenuate rotation induced by methamphetamine and L-dopa in 6-OHDA-treated rats and the hyperactivity induced by acute morphine treatment in mice [59,60]. Based on the results of the influence of uridine on locomotor behavior, we speculated that the changes in locomotor performance in sea cucumbers in the AjK1 treatment group were related to uridine level.

There are 20 kinds of proteinogenic amino acids, among which glycine and serine are non-essential amino acids that can be synthesized by the organism [61]. Glycine and L-serine (one of two forms of serine) are related to several metabolic pathways. Of these pathways, cyanoamino acid metabolism and mineral absorption were significantly inhibited after AjK1 administration. Glycine is known to be inhibitory neurotransmitter in the brainstem and spinal cord [62,63]. Glycinergic interneurons can control motor rhythm generation, and mutations in glycine receptor subunits can cause human motor dysfunction [62]. Blockage of glycine receptors in the spinal cord can result in the loss of the left–right alternating locomotor pattern in newborn mice, but tonic activity increases in neonatal rats [64,65]. The detection of alternating left and right electrophysiological signals in isolated spinal nerve fibers in mice represents a marching movement, called fictive locomotion. According to Kudo et al. (2006), glycine-mediated inhibition was required for the generation of normal fictive locomotion throughout the prenatal stage of rats [66]. Down-regulation or mutations in glycine receptor subunits are also associated with major locomotor deficits in zebrafish [67]. L-serine and its associated metabolism are thought to be essential for several specific functions in the central nervous system, including cell proliferation and intracellular metabolism [68,69]. L-serine is also considered to be a free amino acid essential for exercise [70]. Furthermore, glycine is a possible source of L-serine [68]. In isolated male rats, oral administration of L-serine can decrease locomotor activity in familiar environments and exploratory behavior in novel ones [71]. Additionally, L-serine can suppress motor activity and increase the time-keeping sleeping posture in neonatal chicks under social isolation stress [69]. L-serine also inhibits the abnormal activity induced by beta-methylamino-L-alanine in *D. melanogaster* [72]. In conclusion, both glycine and L-serine can affect locomotor activity. Hence, the changes in L-serine and glycine levels caused by KPs may contribute to the explanation of the locomotor inhibition observed in *A. japonicus* in our experiment.

### 4.3. Possible Functions of the Neuropeptide AjK2

Results of our behavior experiment suggested that AjK2 may not participate in regulating *A. japonicus* locomotor activity, but it still caused changes in muscle metabolites. Among them, the alanine, aspartate, and glutamate metabolism pathway were most significantly affected by the AjK2 treatment. This pathway contains differential metabolites such as aspartate (both L-aspartate and D-aspartate). Aspartate is a nonessential amino acid, and D-aspartate participates in the regulation of sex hormones, which can stimulate the release of gonadotropin-releasing hormone and luteinizing hormone in rats [73]. Exogenous D-aspartate can increase testosterone levels in male animals and affects gametogenesis [74]. Di Fiore et al. (2019) reported that the levels of D-aspartate in wild animals were directly correlated with the periodic levels of sex hormones, suggesting that this amino acid was involved in the regulation of seasonal reproduction [75]. Under the action of AjK2, the higher level of D-aspartate in the longitudinal muscle of *A. japonicus* showed significant changes, which may confirm the premise of Wang et al. (2020) that KPs are related to the seasonal reproduction of *A. japonicus* [7].

## 5. Conclusions

Results of our behavior experiment suggested that AjK1 was more likely to regulate locomotor behavior of *A. japonicus* than AjK2, and that AjK2 seemed to have little effect on it. After the intraperitoneal injection of AjK1, locomotor activity was inhibited, and exercise endurance and movement efficiency decreased. Muscle physiology analysis revealed changes in uridine, glycine, and L-serine levels after AjK1 administration, which may be a potential mechanism responsible for the inhibition of locomotor behavior. Our results suggested that AjK2 is more likely to be related to seasonal reproduction. Further enhancement of AjK2 concentration may be needed to observe its effects on locomotion. More in-depth studies of pharmacokinetics and the underlying biological mechanisms are needed to fill the gaps in our understanding of kisspeptin neuropeptides in invertebrates.

## Figures and Tables

**Table 1:** Experimental groups. Each treatment set had three concentrations.

Concentrations	Control Group	AjK1 Treatment Groups			AjK2 Treatment Groups		
	Seawater	0.1 μM	1 μM	10 μM	0.1 μM	1 μM	10 μM
Groups	S	K1L	K1M	K1H	K2L	K2M	K2H

**Table 2:** Kinetic parameters of KPs acting on the behavior of *A. japonicus*.

Moving Parameters	Kinetic Constants	S	AjK1			AjK2		
			H	M	L	H	M	L
Distance	Maximum reduction (cm)	n.a.	328 ± 34	302 ± 101	n.a.	n.a.	n.a.	n.a.
	Decay rate constant (h-1)	n.a.	0.26 ± 0.04	0.58 ± 0.26	n.a.	n.a.	n.a.	n.a.
	Half-life period (h)	n.a.	2.68 ± 0.40	1.20 ± 0.55	n.a.	n.a.	n.a.	n.a.
Moving duration	Maximum reduction (min)	n.a.	43.5 ± 6.0	46.8 ± 16.3	n.a.	n.a.	n.a.	n.a.
	Decay rate constant (h-1)	n.a.	0.24 ± 0.05	0.61 ± 0.29	n.a.	n.a.	n.a.	n.a.
	Half-life period (h)	n.a.	2.85 ± 0.57	1.13 ± 0.53	n.a.	n.a.	n.a.	n.a.
Velocity	Maximum reduction (cm/min)	n.a.	4.85 ± 0.63	5.07 ± 1.70	n.a.	n.a.	n.a.	n.a.
	Decay rate constant (h-1)	n.a.	0.25 ± 0.05	0.58 ± 0.27	n.a.	n.a.	n.a.	n.a.
	Half-life period (h)	n.a.	2.83 ± 0.54	1.19 ± 0.55	n.a.	n.a.	n.a.	n.a.
Step number	Maximum reduction	n.a.	49.1 ± 6.1	-	-	n.a.	-	-
	Decay rate constant (h-1)	n.a.	0.24 ± 0.04	-	-	n.a.	-	-
	Half-life period (h)	n.a.	2.84 ± 0.51	-	-	n.a.	-	-
Stride	Maximum reduction (cm)	n.a.	4.45 ± 1.06	-	-	2.57 ± 1.02	-	-
	Decay rate constant (h-1)	n.a.	0.28 ± 0.10	-	-	0.75 ± 0.40	-	-
	Half-life period (h)	n.a.	2.46 ± 0.84	-	-	0.92 ± 0.49	-	-
Step frequency	Maximum reduction (step/min)	n.a.	n.a.	-	-	n.a.	-	-
	Decay rate constant (h-1)	n.a.	n.a.	-	-	n.a.	-	-
	Half-life period (h)	n.a.	n.a.	-	-	n.a.	-	-

Based on the first-order kinetics of drug metabolism (Y_after–_Y_before_ = A_max_·e^–kt^), the maximum effect parameter A_max_ and decay rate constant k were obtained by nonlinear regression, and the half-life (τ) was obtained by conversion. The data in the table are mean values ± standard error; n.a. (not available) means that the fitting failed to yield converged results or the fitted parameters were insignificant or negative; –means no nonlinear regression. *R^2^* > 0.6 indicates that the nonlinear regression analysis was reliable.

## Data Availability

The data presented in this study are available in the article. Further information is available upon request from the corresponding author.

## Supplementary Materials

The following supporting information can be downloaded at: https://www.mdpi.com/article/10.3390/ani13040705/s1, Supplementary Figure S1: the schematic of the shooting conditions; Supplementary Figure S2: PCA diagram for quality control; Supplementary Table S1: differential metabolites of treatment groups compared with the control group in positive and negative ion mode.
