# Supplementary material for: Effect of Kisspeptin-Type Neuropeptide on Locomotor Behavior and Muscle Physiology in the Sea Cucumber Apostichopus japonicus
Source: Animals (Basel). 2023 Feb 17;13(4):705. doi: 10.3390/ani13040705 (PMC9951865; doi:10.3390/ani13040705)
Supplement: Supplementary file 1 [file animals-13-00705-s001.zip › animals-1988604-supplementary.pdf]

animals

MDPI

Article

Effect of Kisspeptin-type Neuropeptide on Locomotor Behavior and Muscle Physiology in the Sea Cucumber *Apostichopus japonicus*

Xueying Guo[1,2,3,4,5], Libin Zhang[1,2,3,4,5,6,*], Kang Xiao[7]

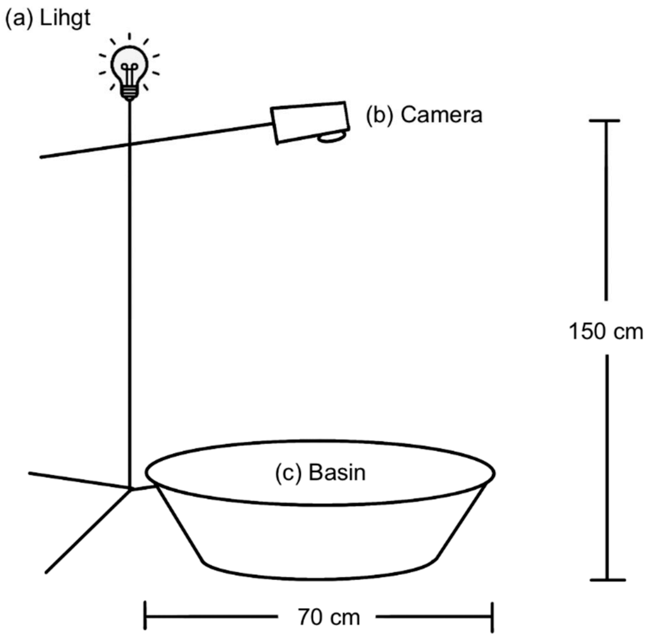

Supplementary Figure 1. The schematic of the shooting conditions. (a) The light source is a 5w bulb. (b) The camera used in the experiment is TLC200 time lapse video camera (Brinno, Taiwan, China). The camera is about 150 cm from the ground. (c) Each experimental sea cucumber was shot independently in a white plastic basin (70 cm in diameter, water line 7 cm).

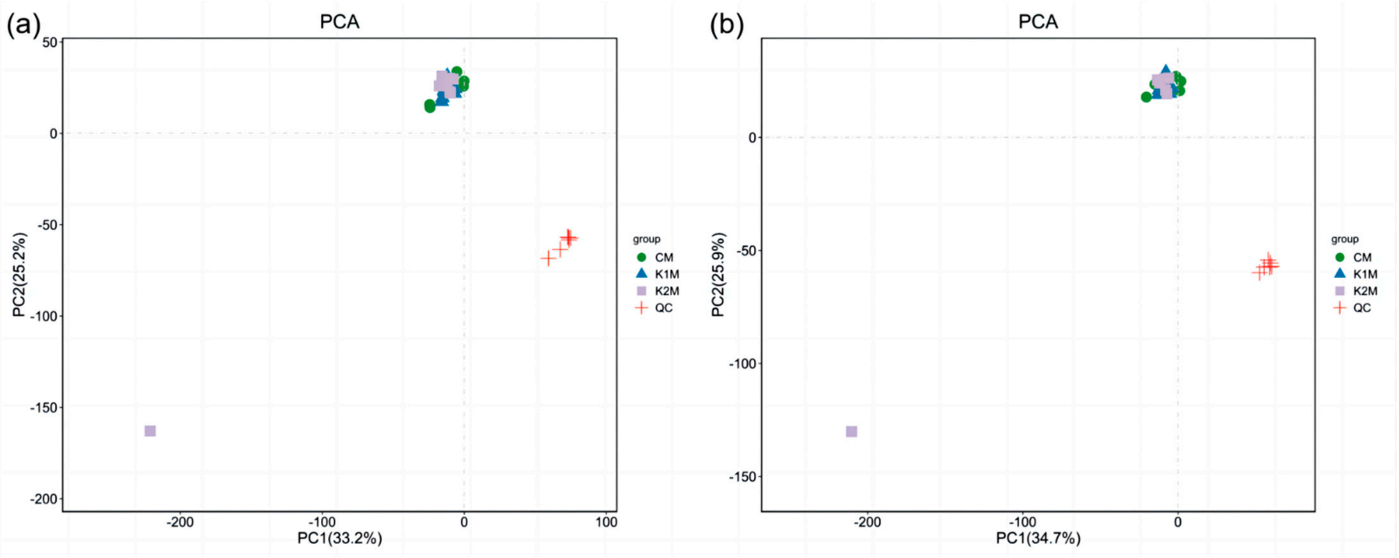

Supplementary Figure 2. PCA diagram for quality control. QC samples are densely distributed, indicating reliable data. (a) Positive ion mode; (b) negative ion mode.

Supplementary Table 1. Differential metabolites of treatment groups (K1M and K2M) compared with the control group in positive and negative ion mode.

Groups	Metabolites	Ion mode	RT (min)	*p*-value	VIP	Regulation
K1M	Aminopyrrolnitrin	POS	361.09	0.05	25.35	down
	alpha-Tocopherol succinate	POS	31.68	0.00	8.94	up
	(+)-Chebulic acid	POS	358.79	0.04	4.13	down
	N8-Acetylspermidine	POS	499.97	0.05	4.04	down
	Ditalimfos	POS	148.18	0.04	3.91	up
	Deoxycytidine	POS	392.23	0.03	3.54	down
	Fosetyl	POS	195.56	0.00	2.81	down
	(3E)-4-(2-Carboxyphenyl)-2-oxobut-3-enoate	POS	302.56	0.04	2.45	up
	erythro-3-Hydroxy-Ls-aspartate	POS	425.81	0.04	2.40	down
	3-Hydroxy-N6,N6,N6-trimethyl-L-lysine	POS	547.64	0.03	2.36	up
	N7-Methylguanosine	POS	99.36	0.04	2.03	up
	PC(18:2(9Z,12Z)/18:0)	POS	101.64	0.04	1.96	up
	Tazobactam	POS	148.13	0.04	1.79	up
	3,3'-Dichlorobenzidine	POS	293.36	0.02	1.78	up
	3,5-Dinitroguaiacol	POS	391.19	0.05	1.73	down

	1-Methyladenosine	POS	147.98	0.05	1.70	up
	alpha-D-Galactopyranuronosyl-(1->4)-alpha-D-galactopyranuronosyl-(1->4)-D-galacturonic acid	POS	31.42	0.00	1.64	up
	Methyl 2-propenyl selenide	POS	286.51	0.00	1.60	down
	Molybdopterinprecursor Z	POS	31.37	0.00	1.46	up
	PC(24:1(15Z)/14:1(9Z))	POS	169.58	0.03	1.20	up
	Chlorophenol red	POS	358.79	0.03	1.19	down
	PE(P-18:1(11Z)/22:5(4Z,7Z,10Z,13Z,16Z))	POS	39.35	0.04	1.15	up
	Luteolin 7-O-[beta-D-glucuronosyl-(1->2)-beta-D-glucuronide]-4'-O-beta-D-glucuronide	POS	169.58	0.00	1.12	up
	Psicofuranine	POS	64.81	0.03	1.12	up
	3-Acetamidobutanal	POS	68.39	0.01	1.03	up
	3,3'-Dihydroxy-4',5,7-trimethoxyflavan	POS	294.49	0.01	1.02	up
	2,4-Dinitroaniline	POS	272.16	0.03	1.02	down
	Norepinephrine sulfate	POS	100.46	0.04	1.00	up
	Palmitoleic acid	NEG	40.66	0.02	13.01	up
	4-Dodecylbenzenesulfonic Acid	NEG	26.97	0.05	7.65	down
	Tectorigenin 4'-sulfate	NEG	38.31	0.04	3.17	up
	3',5'-Cyclic CMP	NEG	30.47	0.00	2.16	up
	Adenine	NEG	269.60	0.05	1.98	down
	Diethylphthalic acid	NEG	62.05	0.04	1.98	down
	L-Serine	NEG	399.11	0.01	1.78	down
	Purine mononucleotide	NEG	38.20	0.02	1.34	up
	Methaneselenol	NEG	147.05	0.04	1.22	up
	Glycine	NEG	398.15	0.05	1.19	up
	2',3'-Cyclic UMP	NEG	30.51	0.00	1.13	up
	[5-(Aminomethyl)furan-3-yl]methyl diphosphate	NEG	360.91	0.04	1.10	down
	2,6-Dichlorophenol-4-(1,4-phthoquinone imine)	NEG	30.51	0.01	1.02	up
	Uridine	NEG	170.12	0.01	1.01	down
K2M	N-Acryloylglycine	POS	395.50	0.00	15.68	down
	DL-Glutamate	POS	409.49	0.04	8.25	down
	2,6-Dimethylpyrazine	POS	359.95	0.01	5.55	down

Turicine	POS	334.83	0.04	4.13	down
2,5-Furandicarboxylate	POS	327.36	0.02	4.06	down
Dihydrozeatin	POS	392.46	0.04	3.93	up
Dichlozoline	POS	237.39	0.03	3.77	down
2-Ethyldihydro-3(2H)-thiophenone	POS	395.45	0.00	3.74	down
Oxamate	POS	367.93	0.02	3.66	down
N8-Acetylspermidine	POS	499.97	0.05	3.26	down
D-Glutamine	POS/NEG	436.47	0.01	2.90	down
3-hydroxy-2-isobutyrate	POS	321.93	0.03	2.75	up
Homogentisate	POS	358.81	0.02	2.53	down
Aniline	POS	261.25	0.03	2.32	down
(2E)-Butenoyl-CoA	POS	58.44	0.04	2.31	down
Valyl-Alanine	POS	399.31	0.02	2.31	down
Aminoacetone	POS	363.19	0.02	2.17	down
2-Azetidinecarboxylic acid	POS	409.52	0.04	2.06	down
(R)-Dihydromaleimide	POS	133.00	0.01	1.82	down
4-Aminohippuric acid	POS	353.03	0.03	1.81	down
S-Acetyldihydrolipoamide	POS	374.90	0.03	1.78	down
(2R)-2-Hydroxy-2-methylbutanenitrile	POS	95.95	0.01	1.58	up
Dihydroxyfumarate	POS	395.50	0.00	1.57	down
S-Prenyl-L-cysteine	POS	363.36	0.04	1.41	up
Chlorophenol red	POS	358.79	0.01	1.37	down
Alanyl-Arginine	POS	447.60	0.05	1.35	down
4,6-Dihydroxy-2-quinolinecarboxylic acid	POS	154.66	0.02	1.32	down
Thiodiglycol	POS	282.99	0.02	1.29	up
Aldosine	POS	308.17	0.02	1.26	down
Methyl 2-propenyl selenide	POS	324.86	0.05	1.19	up
PE-NMe2(18:4(6Z,9Z,12Z,15Z)/20:5(5Z,8Z,11Z,14Z,17Z))	POS	409.59	0.01	1.10	down
1-Pyrrolidinecarboxaldehyde	POS	209.29	0.02	1.09	up
PC(18:4(6Z,9Z,12Z,15Z)/P-18:1(11Z))	POS	409.60	0.01	1.08	down
Chloranocryl	POS	349.59	0.04	1.08	down

5-Aminopentanamide	POS	317.51	0.03	1.00	up
L-Aspartic acid	POS/NEG	416.33	0.00	11.67	down
4-Dodecylbenzenesulfonic Acid	NEG	26.97	0.02	9.48	down
2-phosphonato-D-glycerate(3-)	NEG	362.04	0.04	8.60	down
Pyrrolidonecarboxylic acid	NEG	312.28	0.03	6.53	down
Stearic acid	NEG	38.23	0.04	6.02	down
D-Alanine	NEG	367.80	0.02	5.87	down
gamma-Aminobutyric acid	NEG	408.34	0.01	5.39	down
Pyroglutamic acid	NEG	408.54	0.01	5.12	down
Flupropate	NEG	409.06	0.00	4.06	down
Sarcosine	NEG	416.30	0.00	3.57	down
1-(5-Phospho-D-ribosyl)-5-amino-4-imidazolecarboxylate	NEG	26.89	0.02	2.70	down
D-Malic acid	NEG	438.64	0.04	2.16	down
Fumaric acid	NEG	415.33	0.00	2.05	down
3-Methyl-2-oxovaleric acid	NEG	55.47	0.05	1.86	up
D-Aspartic acid	NEG	438.65	0.01	1.81	down
5-Fluorodeoxyuridine monophosphate	NEG	25.50	0.04	1.79	down
Diethyl phthalic acid	NEG	62.05	0.02	1.47	down
Citric acid	NEG	520.64	0.02	1.41	down
Prostaglandin G2	NEG	51.20	0.01	1.30	down
O-Phosphorylhydroxylamine	NEG	416.32	0.00	1.20	down

Ion mode: positive (POS) or negative (NEG); RT: retention time (min); *p* value: VIP (variable importance in the projection); and regulation trend of the metabolites.
